# Investigation of Isoform Specific Functions of the V-ATPase a Subunit During *Drosophila* Wing Development

**DOI:** 10.3389/fgene.2020.00723

**Published:** 2020-07-10

**Authors:** Dongqing Mo, Yao Chen, Na Jiang, Jie Shen, Junzheng Zhang

**Affiliations:** Department of Entomology and MOA Key Lab of Pest Monitoring and Green Management, College of Plant Protection, China Agricultural University, Beijing, China

**Keywords:** V-ATPase a subunit, wing development, *Drosophila melanogaster*, V-ATPase isoform, V100-2

## Abstract

The vacuolar ATPases (V-ATPases) are ATP-dependent proton pumps that play vital roles in eukaryotic cells. Insect V-ATPases are required in nearly all epithelial tissues to regulate a multiplicity of processes including receptor-mediated endocytosis, protein degradation, fluid secretion, and neurotransmission. Composed of fourteen different subunits, several V-ATPase subunits exist in distinct isoforms to perform cell type specific functions. The 100 kD a subunit (Vha100) of V-ATPases are encoded by a family of five genes in *Drosophila*, but their assignments are not fully understood. Here we report an experimental survey of the *Vha100* gene family during *Drosophila* wing development. A combination of CRISPR-Cas9 mutagenesis, somatic clonal analysis and *in vivo* RNAi assays is used to characterize the requirement of Vha100 isoforms, and mutants of *Vha100-2*, *Vha100-3, Vha100-4*, and *Vha100-5* genes were generated. We show that *Vha100-3* and *Vha100-5* are dispensable for fly development, while *Vha100-1* is not critically required in the wing. As for the other two isoforms, we find that *Vha100-2* regulates wing cuticle maturation, while *Vha100-4* is the single isoform involved in developmental patterning. More specifically, *Vha100-4* is required for proper activation of the Wingless signaling pathway during fly wing development. Interestingly, we also find a specific genetic interaction between *Vha100-1* and *Vha100-4* during wing development. Our results revealed the distinct roles of *Vha100* isoforms during insect wing development, providing a rationale for understanding the diverse roles of V-ATPases.

## Introduction

The vacuolar ATPases (V-ATPases) are ubiquitous proton pumps which play important roles in eukaryotic cells (Forgac, 2007). V-ATPases transport proton into intracellular compartments, and are therefore crucial for pH homeostasis in organelles such as endosomes, secretory vesicles, synaptic vesicles, and lysosomes (Hinton et al., 2009). The V-ATPases are generally required for a broad spectrum of cellular processes, including endosomal trafficking, lysosomal degradation, and exocytosis (Forgac, 2007). Insect V-ATPases are expressed in nearly all epithelial tissues and are well-known for their roles in physiological activities such as secretion of K + and Na + and formation of fluid (Wieczorek et al., 2009). Moreover, recent studies have illuminated the importance of V-ATPases for insect development. In *Drosophila melanogaster*, mutations that dampen the V-ATPases activity are reported to disrupt the formation of eye, wing and egg chambers (Yan et al., 2009; Buechling et al., 2010; Hermle et al., 2010; Vaccari et al., 2010). Further studies demonstrated that V-ATPases are involved in regulation of cell proliferation, cell fate determination and tissue patterning through modification of key developmental signaling pathways (Kobia et al., 2013; Gleixner et al., 2014; Portela et al., 2018; Ren et al., 2018).

The V-ATPase is a large protein complex composed of 14 different subunits that are organized into the cytosolic V1 region and the membrane-bound V0 region (Nelson, 2003). Region V1 hydrolyzes ATP and provides energy to pump protons through the protein lipid pores formed in region V0 (Jianhua et al., 2015). The V1 region contains eight subunits while the V0 domain is assembled by six different subunits (Forgac, 2007). The regulatory C subunit is located in the V1 domain and interacts with subunit a in the V0 domain (Wilkens et al., 2004; Inoue and Forgac, 2005; Cipriano et al., 2008). Therefore, the C subunit is well positioned to control the reversible dissociation of the V-ATPase complex (Inoue et al., 2005; Forgac, 2007). The C subunit is encoded by *Vha44* in *Drosophila*, which is required for endolysosomal acidification and regulates elimination of nurse cells in the ovary (Mondragon et al., 2019), cell competition in the eye imaginal disk (Nagata et al., 2019), and apical endocytosis in the wing disk epithelial cells (Gleixner et al., 2014). Ectopic *Vha44* expression is shown to impair endolysosomal degradation and induce invasive cell behavior in the developing wing disk (Petzoldt et al., 2013) as well as differentiation defects in the eye disk (Portela et al., 2018).

Many of the V-ATPase subunits exist in multiple isoforms which are often expressed in a cell type specific manner (Toei et al., 2010). In *Drosophila*, the V-ATPase multigene family consists of 33 different genes (Julian, 1999; Allan et al., 2005). Apart from five subunits in the V1 region and the accessory subunits VhaAC45 and VhaM8.9, other V-ATPase subunits are encoded by two to five genes (Allan et al., 2005). In vertebrates, isoforms of subunit a in the V0 domain contain information necessary for targeting the V-ATPase complexes to the appropriate plasma membrane (Toyomura et al., 2003; Qi et al., 2007; Saw et al., 2011). In *Drosophila*, the V-ATPase a subunit is encoded by *Vha100-1*, *Vha100-2*, *Vha100-3*, *Vha100-4*, and *Vha100-5* with specific tissue distribution patterns (Toei et al., 2010). Previous studies have suggested that Vha100-1 is an isoform required for synaptic vesicle exocytosis in the nervous system (Hiesinger et al., 2005). Loss of *Vha100-1* leads to vesicle accumulation in synaptic terminals (Wang et al., 2014), neuronal degeneration (Williamson et al., 2010a), and defects in brain wiring (Williamson et al., 2010b). RNAi knock-down experiments indicate that *Vha100-2* is involved in regulation of neural stem cells proliferation (Wissel et al., 2018), acid generation of the midgut (Overend et al., 2016), elimination of nurse cells in the ovary (Mondragon et al., 2019), and cell competition in the eye disk (Nagata et al., 2019). Similar as *Vha100-2*, knock-down of *Vha100-4* also leads to acidification defect in the larval midgut (Overend et al., 2016). The roles of *Vha100-3* and *Vha100-5* are still unclear, and our understanding of whether and how Vha100 isoforms collaboratively regulate the development of specific tissue is incomplete.

In order to further investigate the functional diversity of the V-ATPase a subunit isoforms, we generated and characterized mutants of *Vha100-2, -3, -4*, and *-5*. We found that among the five isoforms, *Vha100-*3 and *Vha100-*5 are dispensable for fly development. We further demonstrated that *Vha100-2* is specifically required for wing cuticle formation, while *Vha100-4* is involved in Wingless signaling activation. Comparative studies revealed that *Vha100-1* and *Vha100-4* execute both independent and redundant function during fly wing development. Our studies uncovered the isoform specific functions of the V-ATPase a subunit during *Drosophila* wing development.

## Materials and Methods

### Fly Genetics

Fly stocks and all fly crosses were maintained at 25°C on standard fly food. The following fly stocks were used: *hh*-Gal4, UAS-*mCD8-gfp/TM6B* (Ren et al., 2018); *Vha100-2* RNAi (TH04790.N; TsingHua Fly Center); *FRT42D*, *Vha44^KG00915^*/*Cyo* (#111534; Kyoto Stock Center); *FRT42D*, *Vha44^*K*05440^*/*Cyo* (#111081; Kyoto Stock Center); *FRT82B, Vha100-1^1^/TM3, Sb* (#39669; Bloomington Drosophila Stock Center); *w;Sco/Cyo* (#2555; Bloomington Drosophila Stock Center); and *w;TM3/TM6B* (#2537; Bloomington Drosophila Stock Center). The *Ubx-FLP; FRT82B, Ubi-RFP/TM6B, Ubx-FLP; FRT42D, Ubi-RFP/Cyo*, and *Ubx-FLP; FRT42D, Ubi-GFP*/*Cyo* stocks were used to generate mosaic mutant clones in the wing disks (Ren et al., 2018). The *sens−GFP* reporter was described before (Sarov et al., 2016) and obtained from Bloomington Drosophila Stock Center (#38666). The *fz3*-LacZ reporter was described before (Sato et al., 1999).

### CRISPR-Cas9 Mediated Mutagenesis

The sgRNA targets were designed against the genomic sequences of *Vha100-2, Vha100-3, Vha100-4*, and *Vha100-5* with CRISPR Optimal Target Finder^1^ (Gratz et al., 2014). Templates for sgRNA transcription were generated by annealing of two DNA oligonucleotides and subsequent PCR amplification (Bassett et al., 2013). *In vitro* transcription was performed with the T7 RiboMAX^TM^ Kit (Promega, P1320) and the sgRNAs were purified by phenol-choloroform extraction and isopropanol precipitation. Cas9 mRNA was transcribed with the mMESSAGE mMACHINE^®^ T7 Transcription Kit (Ambion), using a linearized plasmid containing the Cas9 cDNA (Addgene plasmid 42251) as template. The Cas9 mRNA were polyadenylated with the Escherichia *coli* Poly(A) polymerase Kit (NEB), and purified with the RNeasy Mini Kit (QIAGEN). 15 μg of Cas9 mRNA and 7.5 μg sgRNA were mixed with DEPC water in a 30 μl volume for embryo injection. Fly embryos of the *w*^1118^ strain (#5905; Bloomington Drosophila Stock Center) were injected using standard protocols by Fungene Biotech (Beijing, China).

Males developed from the injected embryos (G0) were outcrossed to virgin females of *TM3/TM6B* (for *Vha100-2* and *Vha100-4*) or *Sco/Cyo* (for *Vha100-3* and *Vha100-5*). Single G1 males were each crossed to 4–5 females of the corresponding balancer stocks and the progenies (G2) bearing the same balancer chromosome were maintained as an independent stock. About 50 G2 stocks were established from each injection, and mutations were screened by PCR test of genomic DNAs. Primer pairs were designed to generate PCR products covering the target sites, which were compared with the control sequences amplified from *w*^1118^ genomic DNA. The primers used are: V100-2F: AACGTTGTCGTTGGCTGAAGCA; V100-2R: ATGT CATCCTGGATGTCGGGCA; V100-3F: CTGCGCATCGTGG ACAGTCTG; V100-3R: GAATGATCAGGTTGAAGGAGC; V100-4F: GCTGTGCTCCGAAAGTGAG; V100-4R: ACCT TGTGACCCTCCTTGTT; V100-5F: CAGTTATAACACTCG ATTTGA; and V100-5R: TTGAGTTCTTGCATGTGCCGGA. Mutant alleles were identified and named as *Vha100-2^*D*2^, Vha100-3^*D*3^*, *Vha100-4^*D*4^*, and *Vha100-5^*D*5^*. FRT82B (#2035; Bloomington Drosophila Stock Center) was recombined into the *Vha100-2^*D*2^* and *Vha100-4^*D*4^* mutant genomes by standard genetic crosses for further mosaic analysis.

### mRNA *in situ* Hybridization in *Drosophila* Wing Imaginal Disks

The coding regions of *Vha100-2* (1208 bp–1430 bp of GeneBank #AAF55551) and *Vha100-4* (982 bp–1240 bp of GeneBank #AAF55550) were used to generate antisense RNA probes for *in situ* hybridization. An autofluorescent alkaline phosphatase substrate (Vector) was used to visualize mRNA in the rhodamine channel, and mutant clones were marked by immunofluorescence staining of GFP protein as described before (Su et al., 2011).

### Immunofluorescence Staining

Wing disks dissected from third-instar larvae were fixed in 4% paraformaldehyde for 15 min, blocked in PBS containing 0.1% Triton X–100 and 0.2% BSA for 1 h, and incubated overnight at 4°C with the following primary antibodies: mouse anti-Cut (1:100; 2B10; and DSHB), mouse anti-Wingless (1:200; 4D4; and DSHB), mouse anti-Notch intracellular domain NICD (1:200; C17.9C6; and DSHB), mouse anti- Notch extracellular domain NECD (1:200; C458.2H; and DSHB), mouse anti-Dl (1:200; C594.9B; and DSHB), rabbit anti-GFP (1:2000; A11122; and Thermo Fisher), and rabbit anti-Caspase3 (1:200; Cell signaling). Alexa fluor-conjugated secondary antibodies (1:400; Invitrogen) were used. Alexa fluor-568 conjugated phalloidin was used to label cell morphology (1:200; Thermo Fisher). The fluorescence images were acquired with Leica SP8 confocal microscope and processed in Photoshop and ImageJ.

### Eosin Y and FB28 Staining and Microscopy of the Adult Wing

Adult flies with correct genotypes were collected and fixed overnight in isopropanol. Dissected adult wings were mounted in Euparal mounting media (BioQuip). For Eosin Y and FB28 staining experiments, 2 days old files were fixed in formaldehyde phosphate buffer, washed several times with PBS, and stained with FB28 solution (1 mg/ml; Sigma-Aldrich) at room temperature for 1 h or 0.5% Eosin Y at 55°C for 35 min. Adult wings were then dissected and mounted in 80% glycerol. The images were captured with a Leica DMIL inverted microscope equipped with a QICAM Fast 1394 digital camera.

## Results

### Vha100 Isoforms Are Differentially Required for Fly Development

**FIGURE 1:**
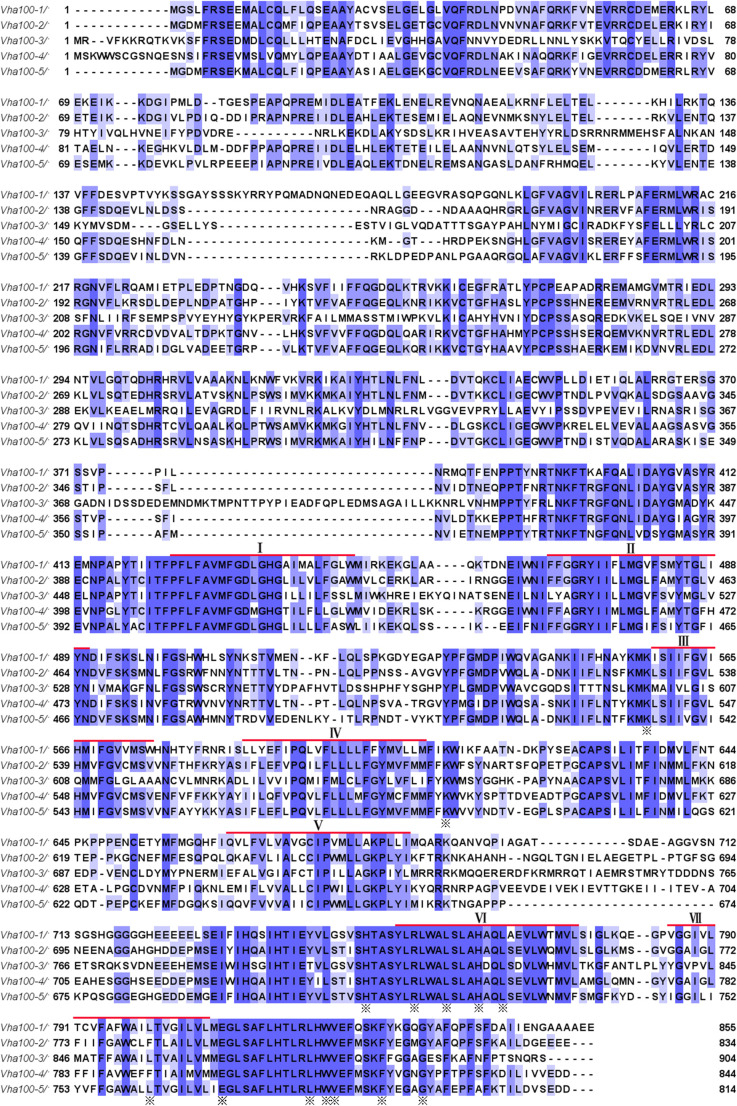
Amino acid sequences and structures of V-ATPase subunit a isoforms in *Drosophila*. Alignment of the five *Drosophila* V-ATPase a subunit isoform sequences (Vha100-1, Vha100-2, Vha100-3, Vha100-4, and Vha100-5) is shown. Identical residues are indicated by blue shades and similar residues are labeled by light purple shades. The seven putative transmembrane helices predicted from topographical analysis are shown with a red bar. The residues whose mutation has a significant effect on activity or assembly of the V-ATPase are indicated by asterisks. GenBank accession numbers assigned to Vha100-1, Vha100-2, Vha100-3, Vha100-4, and Vha100-5 are AAF56861, AAF55551, AAM68427, AAF55550, and AAF53116, respectively.

The a subunit of the V-ATPase is encoded by five genes in *Drosophila*, which are spread at different locations throughout the genome (Supplementary Figures S1A–D). The -1, -2, -3, -4, and -5 isoform comprises 855, 834, 904, 844, and 814 amino acid residues, respectively. A hidden Markov model (Krogh et al., 2001; http://www.cbs.dtu.dk/services/TMHMM-2.0/) predicated that these isoforms have closely similar structures with seven putative transmembrane regions, which are conserved among the five isoforms (Figure 1). High conservation is also evident for the hydrophilic amino and hydrophobic carboxy terminals of fly Vha100 isoforms (Figure 1). Detailed analysis of sequence homology reveals that Vha100-3 is the most diverse member of the family, while the other isoforms share a similarity about 70% between each other (Table 1). A number of residues of the a subunit have been experimentally demonstrated to be important for the activity or assembly of V-ATPase complex in yeast (Leng et al., 1996, 1998), which were later found to be conserved in mouse orthologs (Nishi and Forgac, 2000; Toyomura et al., 2000). All of these residues are conserved in the fly isoforms, with the exception of L800 (the Vha100-1 numbering), which is conserved in -3 and -5 but is a phenylalanine in -2 and -4 (Figure 1). It is likely that the less conserved regions may render different functions of Vha100 isoforms. Construction of a phylogenetic tree (Minh et al., 2013; Trifinopoulos et al., 2016) using the mouse, fly and yeast sequences reveals that the development of multiple isoforms of the a subunit appears to have occurred independently in these species (Supplementary Figure S1E).

**TABLE 1:** The sequence identity (and similarity) between pairs of *Drosophila* a subunit isoforms.

Pairs of isoforms	Identity and (similarity)	Pairs of isoforms	Identity and (similarity)
Vha100-1 and Vha100-2	58% (71%)	Vha100-2 and Vha100-4	65% (79%)
Vha100-1 and Vha100-3	36% (51%)	Vha100-2 and Vha100-5	65% (79%)
Vha100-1 and Vha100-4	53% (67%)	Vha100-3 and Vha100-4	33% (51%)
Vha100-1 and Vha100-5	57% (71%)	Vha100-3 and Vha100-5	35% (53%)
Vha100-2 and Vha100-3	35% (54%)	Vha100-4 and Vha100-5	56% (72%)

**FIGURE 2:**
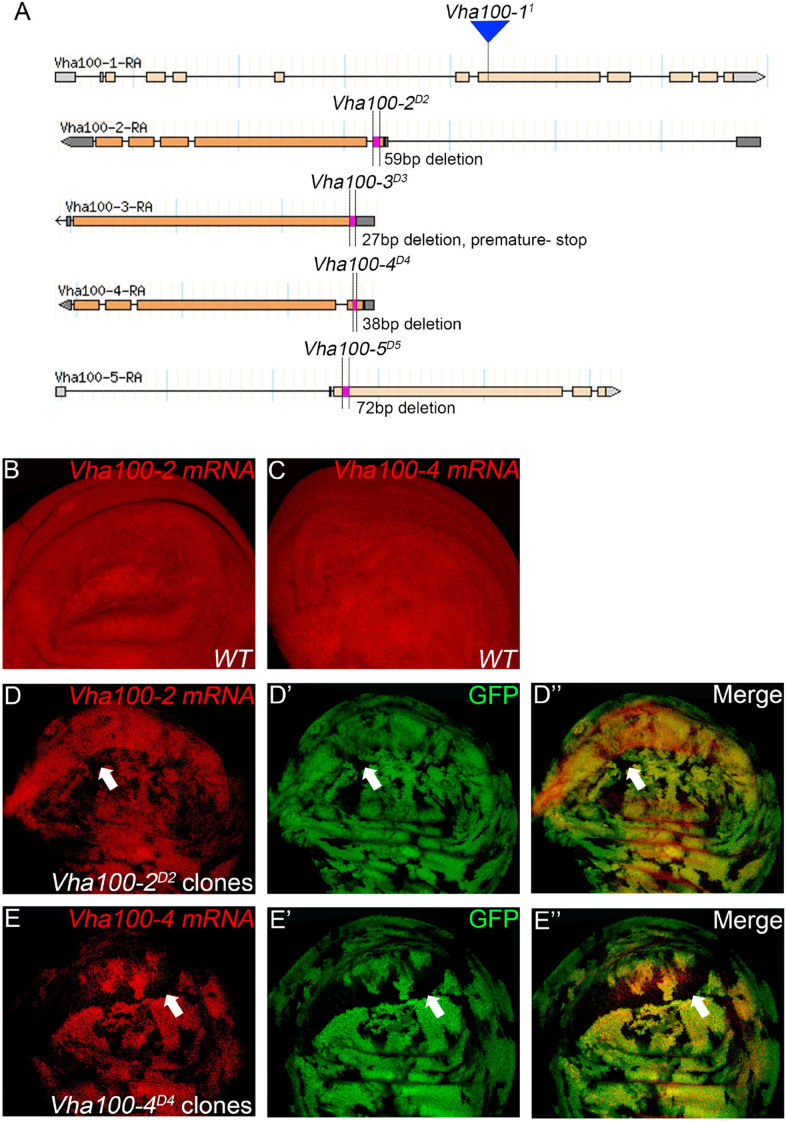
Generation and verification of *Vha100* mutants. **(A)** Schematic illustration of *Vha100* mutant alleles. The *Vha100-1^1^* is a loss of function allele caused by mutation in the splice acceptor site. CRISPR-Cas9 mediated deleterious alleles are generated for other members of the *Vha100* family. The position and molecular nature of each allele is labeled. **(B–E)** *In situ* hybridization in *Drosophila* wing imaginal disks. In wild type wing disks, *Vha100-2* **(B)** and *Vha100-4* **(C)** are ubiquitously expressed. In *Vha100-2^*D*2^* mutant clones, the mRNA levels (red) are significantly down-regulated **(D)**. The mRNA levels of *Vha100-4* (red) are significantly down-regulated in *Vha100-4^*D*4^* clones **(E)**. The down-regulation of *Vha100-2* (*n* = 11) and *Vha100-4* (*n* = 14) in mutant clones is fully penetrant. The mutant clones are marked by absence of GFP. Representative mutant clones are indicated by arrows. Scale bars: 10 μm.

Previous studies have isolated several *Vha100-1* mutant alleles and revealed a neuronal specific role of *Vha100-1* during fly development (Hiesinger et al., 2005). For the other four isoforms, mutagenesis analyses have not been reported yet. To better understand how Vha100 isoforms function in different developmental contexts, we generated *Vha100-2*, *Vha100-3*, *Vha100-4*, and *Vha100-5* mutants by CRISPR-Cas9 mediated genome editing (Table 2). Mutations with small deletions were identified by genomic DNA PCR (Supplementary Figure S2) and used for further analysis (Figure 2A). Homozygous *Vha100-3^*D*3^* and *Vha100-5^*D*5^* mutant flies are viable and fertile with normal appearance of body structure and tissue morphology. Previous studies have shown that the expression of *Vha100-3* is restricted to the testes in adult males, while the mRNA of *Vha100-5* is undetectable by *in situ* hybridization in fly larvae (Wang et al., 2004; Allan et al., 2005). We reason that these two isoforms might perform specific physiological roles, but are dispensable for fly development. Therefore, we moved on to examine the roles of *Vha100-1*, *Vha100-2*, and *Vha100-4* in fly wing development.

**TABLE 2:** Summary of CRISPR-Cas9 sgRNA sequences.

Gene symbol	sgRNA-1 sequence	sgRNA-2 sequence
*Vha100-2*	GATGTTCCGTAGTGAGGAGA	GTATACCTCCGTATCTGAGC
*Vha100-3*	GATTTCGACGACTGTCCAGT	GCACAGCTTCGCTTTGAACA
*Vha100-4*	GCAAATGTATCTGCAGCCGG	GTTGCCGCCTTGGGCGAGGT
*Vha100-5*	GGGCGTAGGCTGCCTCCGGC	GTTTCGCGATCTGAACGAGG

As *Vha100-1^1^* (Hiesinger et al., 2005), *Vha100-2^*D*2^*, and *Vha100-4^*D*4^* are homozygous lethal mutants, somatic mosaic clones were generated using the FLP-FRT system to examine their roles in wing development. The FLP recombinase catalyzes exchange of the homologous chromosome arms and induces the generation of homozygous mutant cell clones with randomized size and location (Xu and Rubin, 1993). Importantly, the wild type cells surrounding the mutant clones serve as rigorous internal control for developmental studies (Theodosiou and Xu, 1998). Taking advantage of the FLP-FRT system, we evaluated whether *Vha100-2^*D*2^* and *Vha100-4^*D*4^* (Figure 2A) behave as loss-of-function alleles by examining their mRNA levels in mutant mosaic clones. Both *Vha100-2* (Figure 2B) and *Vha100-4* (Figure 2C) were ubiquitously expressed in wing disk cells. Compared with the neighboring wild type cells, the expression level of *Vha100-2* were significantly down-regulated in *Vha100-2^*D*2^* homozygous cells (Figure 2D). The expression of *Vha100-4* were also obviously decreased in *Vha100-4^*D*4^* clones (Figure 2E).

### Vha100-2 Is Specifically Required for Wing Cuticle Formation

We first examined the overall requirement of V-ATPase activity during wing development, and the contributions of distinct Vha100 isoforms were further dissected. Two mutants of the regulatory C subunit *Vha44* were tested (Supplementary Figure S3A) and both of them resulted in various developmental defects including loss of wing marginal bristles and nicking of wing margin (Supplementary Figures S3B–D). We then investigated whether *Vha44* regulates Notch (N) and Wingless (Wg) signaling activity, two major signaling pathways that were affected by mutations of other V-ATPase subunits (Vaccari et al., 2010; Kobia et al., 2013; Gleixner et al., 2014; Portela et al., 2018; Ren et al., 2018). In the wild type imaginal wing disk, the N target gene *cut* is produced in a narrow stripe of cells along the dorsal/ventral (D/V) boundary (Supplementary Figure S3E). In *Vha44^*K*05440^* mutant clones, the expression of Cut is abolished when the clones are located at the D/V boundary (Supplementary Figure S3F). Clones of *Vha44* mutant cells also displayed various degrees of accumulation of the ligand molecule Delta (Dl) and N protein itself, which were detected as intracellular puncta (Supplementary Figures S3G–J). During fly wing development, the Wg protein is generated at the D/V boundary (Supplementary Figure S3K) and transported throughout the wing disk to activate down-stream targets (Swarup and Verheyen, 2012). In *Vha44* mutant cells, the expression level of Wg was not significantly affected, while the distribution of Wg protein in signal receiving cells was altered (Supplementary Figure S3L). As a consequence, the activity of Wg signaling was dampened in *Vha44* mutant cells (Supplementary Figures S3M,N), which was monitored by a *fz3*-LacZ reporter (Sato et al., 1999). In addition, we found that *Vha44^*K*05440^* mutant cells displayed aberrant cellular cortex morphology (Supplementary Figures S3O,P) and likely underwent apoptosis (Supplementary Figures S3Q,R).

**FIGURE 3:**
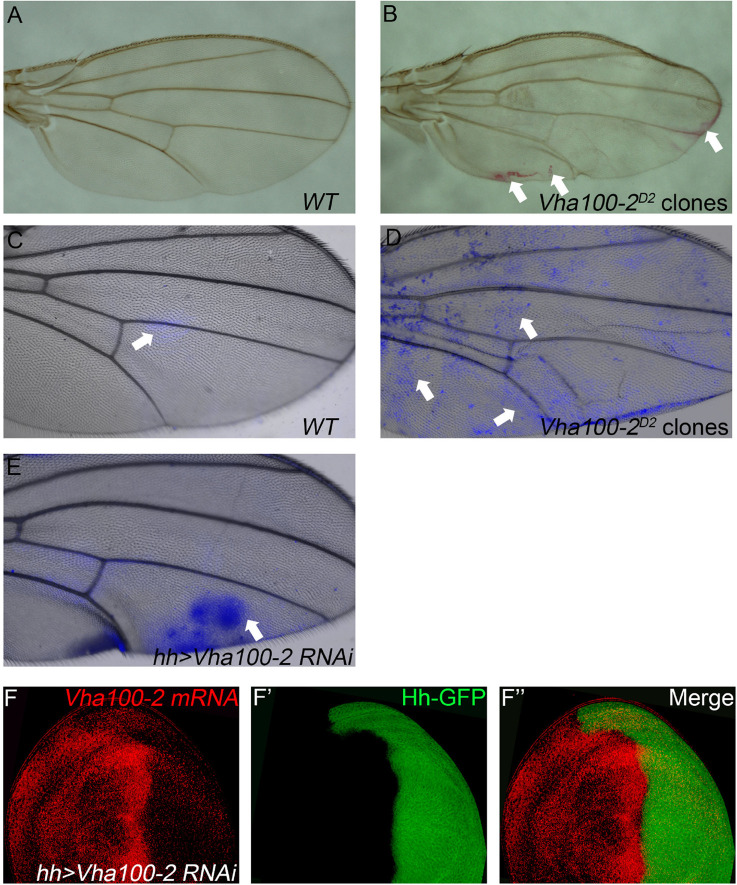
*Vha100-2* is specifically required for wing cuticle formation. **(A,B)** Penetration of Eosin Y through the wing cuticle. The adult wing of *Ubx-FLP; FRT82B, Ubi-RFP* stock is used as wild type control, which is resistant to Eosin Y penetration **(A)**. By contrast, patches with Eosin Y staining (red) are observed in fly wings harboring homozygous *Vha100-2^*D*2^* mutant clones **(B)**. **(C–E)** Penetration of FB28 through the wing cuticle. In wild-type control wings, the FB28 dye (blue) shows limited penetration competency **(C)**. The FB28 dye (blue) scatters into the wing bearing homozygous *Vha100-2^*D*2^* clones **(D)**. In *Vha100-2* RNAi expression area, the penetration of the FB28 dye (blue) is evident **(E)**. **(F)** The mRNA level of *Vha100-2* (red) are down-regulated in RNAi expressing cells (marked by GFP). Representative Eosin Y and FB28 staining patches are indicated by arrows. The down-regulation of *Vha100-2* in RNAi expressing region is fully penetrant (*n* = 7). Scale bars: 0.5 mm in **(A–E)** and 10 μm in **(F)**.

In contrast to the *Vha44* mutants, fly wings harboring homozygous *Vha100-2^*D*2^* mutant clones displayed wrinkles in the wing surface, without disruption of the vein patterns and wing margin integrity (Figures 3A,B). In agreement with the lack of patterning defects in the adult wing, the expression of Cut remained unaltered in *Vha100-2^*D*2^* mutant clones (Supplementary Figure S4A). The expression level and distribution of Wg, N, and Dl proteins were also normal in *Vha100-2^*D*2^* mutant cells (Supplementary Figures S4B–D), suggesting that Vha100-2 is not required for these two pathways. Furthermore, phalloidin staining showed that *Vha100-2^*D*2^* mutant had little influence for cell morphology (Supplementary Figure S4E). Wrinkling of the wing surface is often associated with defective cuticle deposition (Moussian, 2010). To further characterize the role of *Vha100-2* on wing cuticle formation, we analyzed the cuticle impermeability by FB28 and Eosin Y staining (Wang et al., 2016, 2017). Wild-type wings with an intact cuticle layer is almost resistant to Eosin Y penetration (Figure 3A). By contrast, wings harboring homozygous *Vha100-2^*D*2^* mutant clones showed patches of Eosin Y staining (Figure 3B). Similarly, a polysaccharide-specific dye FB28 was able to penetrate through the cuticle of wings bearing *Vha100-2^*D*2^* mutant clones (Figures 3C,D). We further confirmed the role of *Vha100-2* in cuticle integrity by knock-down of *Vha100-2* expression in the posterior compartment of the wing. Regional specific inhibition of *Vha100-2* led to wrinkles in the RNAi expressing area as well as diffusion of the FB28 dye (Figure 3E). Consistent with the adult wing phenotype, RNAi knock-down led to attenuation of *Vha100-2* mRNA level when examined in the wing disk (Figure 3F). Taken together, our results suggest that *Vha100-2* might be involved in cuticle formation of the wing.

### Vha100-4 Is Required for Proper Activation of the Wg Signaling Pathway

**FIGURE 4:**
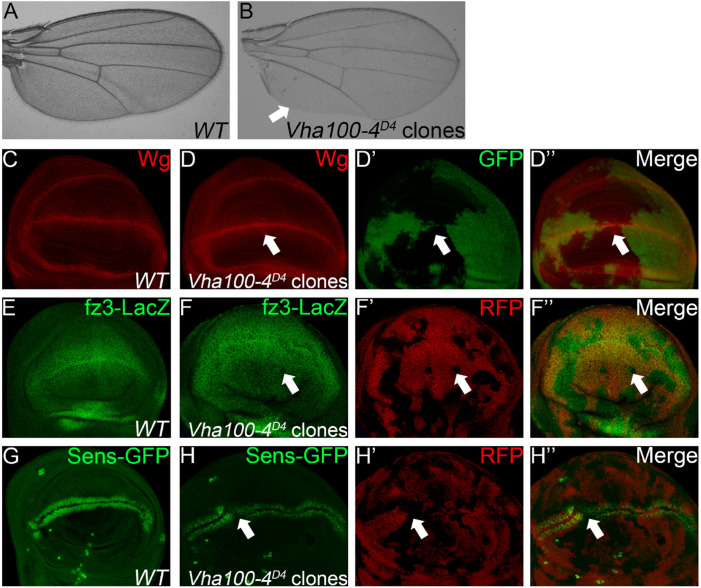
*Vha100-4* is required for proper activation of the Wg signaling pathway. **(A,B)** The adult wing of *W*^1118^ stock is used as wild type control **(A)**. Wing margin notches and marginal bristle loss are observed in homozygous *Vha100-4^*D*4^* mutant clones **(B)**. **(C,D)** Immunostaining shows that Wg (red) is expressed by cells at the D/V boundary in the pouch of wild type wing disk **(C)**. Wg proteins (red) are accumulated in a subset of mutant *Vha100-4^*D*4^* clones (*n* = 7/13; **D**). **(E,F)** The Wg signaling reporter *fz3-LacZ* (green) is expressed by all cells in the pouch area of wing disk **(E)**. Expression of *fz3-LacZ* (green) is dampened in *Vha100-4^*D*4^* mutant clones (*n* = 6/11; **F**). **(G,H)** Sens-GFP (green) is expressed in two rows of cells which are above and below the D/V boundary **(G)**. Expression of the *Sens-GFP* reporter (green) was inhibited in a subset of *Vha100-4^*D*4^* mutant clones (*n* = 7/12; **H**). The mutant clones are marked by absence of GFP **(D)** or RFP **(F,H)**. Representative mutant clones are indicated by arrows. Scale bars: 0.5 mm in **(A,B)** and 10 μm in **(C–H)**.

When *Vha100-4^*D*4^* mutant clones were induced in the wing, marginal notches and bristle loss were observed (Figures 4A,B). These marginal defects are typically resulted from disruption of N or Wg signaling activity (Logan and Nusse, 2004). Interestingly, we found that neither the target Cut nor the signaling molecules (N and Dl) were changed in *Vha100-4^*D*4^* mutant cells (Supplementary Figure S5). On the other hand, aggregation of Wg proteins were observed in some mutant cells (Figures 4C,D), and the expression of the Wg activity reporter *fz3*-LacZ (Figures 4E,F), and *sens-*GFP (Figures 4G,H) were dampened in a subset of *Vha100-4^*D*4^* mutant clones. Collectively, these findings provide evidence that Vha100-4 is likely involved in Wg signaling activation during fly wing development.

### Vha100-1 Performs Redundant Function With Vha100-4

**FIGURE 5:**
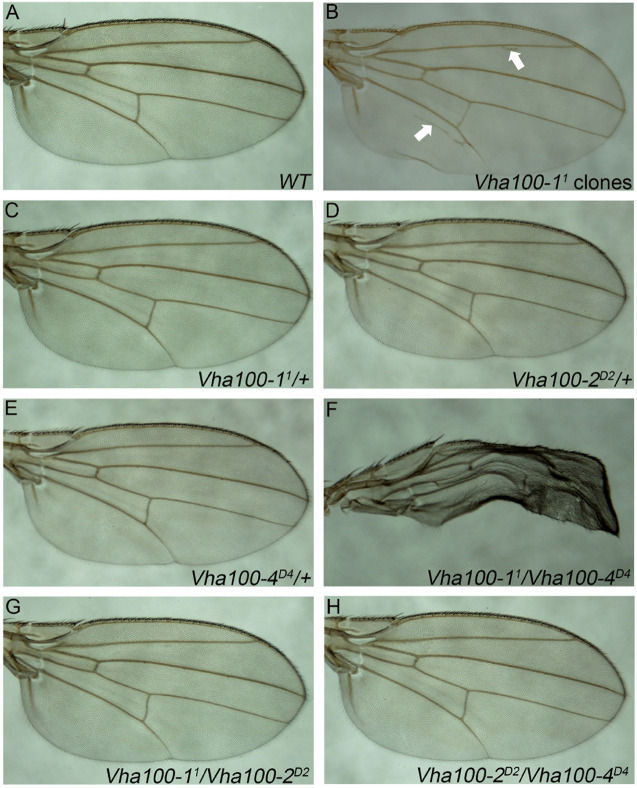
*Vha100-1* and *Vha100-4* perform redundant functions during fly wing development. **(A,B)** The adult wing of *W*^1118^ stock is used as wild type control **(A)**. Ectopic veins (marked by arrows) are formed in wings bearing *Vha100-1^1^* mutant clones **(B)**. **(C–H)** Compensation tests among *Vha100-1*, *Vha100-2*, and *Vha100-4*. The adult wings of heterozygous *Vha100-1^1^* **(C)**, *Vha100-2^*D*2^* **(D)**, and *Vha100-4^*D*4^* **(E)** flies are indistinguishable from wild type controls. *Trans*-heterozygous *Vha100-1^1^/Vha100-4^*D*4^* flies show severe wing developmental defects **(F)**. Wings of *Vha100-2^*D*2^*/*Vha100-1^1^* **(G)** and *Vha100-2^*D*2^*/*Vha100-4^*D*4^* **(H)** flies display normal developmental patterns. Scale bars: 0.5 mm.

Although previous studies suggest that Vha100-1 functions in the neuronal system to regulate fly development (Hiesinger et al., 2005; Wang et al., 2014), we noticed that ectopic veins were formed in wings bearing *Vha100-1^1^* mutant clones (Figures 5A,B). However, N and Wg signaling were not affected in *Vha100-1^1^* mutant cells (Supplementary Figures S6A,B). In addition, we noticed that *Vha100-1^1^* mutant cells did not undergo apoptosis (Supplementary Figure S6C) and displayed normal cell morphology (Supplementary Figure S6D). The overall patterning and wing margin integrity was not affected by *Vha100-1^1^* mutant clones, suggesting that Vha100-1 might function in specific processes during vein cell differentiation.

Mutations of *Vha100-1*, *Vha100-2*, and *Vha100-4* were unable to fully recapture the wing defects caused by *Vha44* mutant, despite that they are all essential genes required for normal development. These results indicate that these isoforms may function redundantly and the loss of single isoform can be compensated by the remaining family members in certain developmental contexts. To test this hypothesis, we crossed *Vha100-1^1^*, *Vha100-2^*D*2^*, and *Vha100-4^*D*4^* flies with each other and examined the wing phenotypes in the *trans*-heterozygous progenies. For each mutant allele, the heterozygotes were indistinguishable with wild type controls (Figures 5C–E). The wings of *Vha100-1^1^/Vha100-4^*D*4^* flies were severely malformed (Figure 5F), indicating a strong genetic interaction and functional redundancy between *Vha100-1* and *Vha100-4*. Combination of *Vha100-2^*D*2^* with either *Vha100-1^1^* (Figure 5G) or *Vha100-4^*D*4^* (Figure 5H) shows no effect on fly development and wing morphology, suggesting that Vha100-2 likely functions independently of the other two isoforms.

## Discussion

The V-ATPases are highly conserved multi-subunit pumps that transport hydrogen ions in exchange for ATP. Present in the endo-membranes of all eukaryotic cells, they are well known regulators for acidification of various intracellular compartments (Forgac, 2007). Studies in the larval midgut of the tobacco hornworm *Manduca sexta* are among the first reports to reveal that V-ATPases also pump protons across the plasma membranes of many specialized animal cells (Wieczorek et al., 2009). The great diversity of functions that the V-ATPases serve in eukaryotic organisms is recognized as a remarkable feature (Forgac, 2007; Wieczorek et al., 2009). Several subunits of the V-ATPases are encoded by multiple isoforms, and tissue specific expression of different isoforms have been demonstrated as a common strategy to fulfill the diverse requirement for V-ATPases (Toei et al., 2010). However, whether different isoforms are used in the same tissue to execute distinct functions is still an open question.

Here we report that isoforms of the V-ATPase a subunit are differentially required for *Drosophila* wing development. We provide genetic evidence that three of the five Vha100 isoforms (*Vha100-1*, *Vha100-2*, and *Vha100-4*) are involved in fly wing development. Somatic clonal analysis show that *Vha100-1* regulates vein formation, while *Vha100-2* functions in cuticle deposition and *Vha100-4* participates in Wg signaling transduction. It is likely that *Vha100-1* regulates vein formation through pathways transduced by other signaling molecules, such as bone morphogenetic protein (BMP), and epidermal growth factor (EGF) during fly wing development (Blair, 2007). Our results strongly support a model that V-ATPases might exist as multiple subtypes composed by diverse subunit isoforms in the same cells to meet the diversified demands. The accumulation of signaling molecules and alteration of signaling activities are not always fully penetrant in *Vha100* mutant cells, which is also noticed in previous studies on other V-ATPase subunits (Yan et al., 2009; Vaccari et al., 2010; Ren et al., 2018). During tissue development, the V-ATPase regulates acidification of cellular organelles which are necessary for protein sorting, trafficking, and turnover (Forgac, 2007). It is conceivable that disrupting V-ATPase activity results in aberrant trafficking and degradation of signaling molecules, a highly dynamic process that might be only partially captured by conventional genetic analysis. In addition, our compensation tests suggest that beyond their specialized roles, *Vha100-1*, and *Vha100-4* likely function redundantly for wing patterning and growth. This redundancy may also help to explain the minor defects observed when *Vha100-1* and *Vha100-4* single mutant clones were induced in the wing. Our observations suggest that the V-ATPases subtypes are able to constitute a coordinated network in cells. Further studies are required to dissect the exact mechanisms underlying the mode of action for V-ATPases.

Previous studies using RNAi knock-down experiments indicate that *Vha100-2* is involved in the development of multiple fly tissues (Wang et al., 2014; Overend et al., 2016; Mondragon et al., 2019; Nagata et al., 2019). We find that *Vha100-2* is the sole isoform required for wing cuticle integrity. This is the first report regarding a role of V-ATPases in insect cuticle formation. The cuticle is a multifunctional tissue that covers the whole body of insects, which provides significant protections from life threatening dangers such as dehydration, predators and pathogen infection (Moussian, 2010). It is possible that V-ATPases might regulate membrane trafficking and secretion of the cuticle components in wing epithelia cells in a similar fashion as reported in *Caenorhabditis elegans* (Liégeois et al., 2007). However, V-ATPases may as well function in other steps during cuticle formation, which needs to be further explored.

## Data Availability Statement

The raw data supporting the conclusions of this article will be made available by the authors, without undue reservation, to any qualified researcher.

## Author Contributions

DM performed the mosaic screen and identified the *Vha44* mutants. DM, YC, and NJ performed the immunostaining analyses. JZ performed the mRNA *in situ* hybridization. DM, YC, NJ, and JZ performed the statistical analyses for wing phenotypes. DM prepared the original draft. JS and JZ reviewed and edited the manuscript. JS and JZ supervised the project. All authors approved the final manuscript submitted for publication.

## Conflict of Interest

The authors declare that the research was conducted in the absence of any commercial or financial relationships that could be construed as a potential conflict of interest.

## Footnotes

1 http://tools.flycrispr.molbio.wisc.edu/targetFinder/

## References

[B1] Allan A. K. Juan D. Davies S. A. Julian A. T. D. (2005). Genome-wide survey of V-ATPase genes in *Drosophila* reveals a conserved renal phenotype for lethal alleles. *Physiol. Genom.* 22 128–138. 10.1152/physiolgenomics.00233.2004 15855386

[B2] Bassett A. R. Tibbit C. Ponting C. P. Liu J. L. (2013). Highly efficient targeted mutagenesis of *Drosophila* with the CRISPR/Cas9 system. *Cell Rep.* 4 220–228. 10.1016/j.celrep.2013.06.020 23827738 PMC3714591

[B3] Blair S. S. (2007). Wing vein patterning in *Drosophila* and the analysis of intercellular signaling. *Annu. Rev. Cell Dev. Biol.* 23 293–319. 10.1146/annurev.cellbio.23.090506.123606 17506700

[B4] Buechling T. Bartscherer K. Ohkawara B. Chaudhary V. Spirohn K. Niehrs C. (2010). Wnt/Frizzled signaling requires dPRR, the *Drosophila* homolog of the prorenin receptor. *Curr. Biol.* 20 1263–1268. 10.1016/j.cub.2010.05.028 20579883

[B5] Cipriano D. J. Wang Y. Bond S. Hinton A. Jefferies K. C. Qi J. (2008). Structure and regulation of the vacuolar ATPases. *Biochim. Biophys. Acta* 1777 599–604. 18423392 10.1016/j.bbabio.2008.03.013 PMC2467516

[B6] Forgac M. (2007). Vacuolar ATPases: rotary proton pumps in physiology and pathophysiology. *Nat. Rev. Mol. Cell Biol.* 8 917–929. 10.1038/nrm2272 17912264

[B7] Gleixner E. M. Canaud G. Hermle T. Guida M. C. Kretz O. Huber T. B. (2014). V-ATPase/mTOR signaling regulates megalin-mediated apical endocytosis. *Cell Rep.* 8 10–19. 10.1016/j.celrep.2014.05.035 24953654

[B8] Gratz S. J. Ukken F. P. Rubinstein C. D. Thiede G. Donohue L. K. Cummings A. M. (2014). Highly specific and efficient CRISPR/Cas9-catalyzed homology-directed repair in *Drosophila*. *Genetics* 196 961–971. 10.1534/genetics.113.160713 24478335 PMC3982687

[B9] Hermle T. Saltukoglu D. Grucnewald J. Walz G. Simons M. Gru J. (2010). Regulation of Frizzled-dependent planar polarity signaling by a V-ATPase subunit. *Curr. Biol.* 20 1269–1276. 10.1016/j.cub.2010.05.057 20579879

[B10] Hiesinger P. R. Fayyazuddin A. Mehta S. Q. Rosenmund T. Schulze K. L. Zhai R. G. (2005). The v-ATPase V0 subunit a1 is required for a late step in synaptic vesicle exocytosis in *Drosophila*. *Cell* 121 607–620. 10.1016/j.cell.2005.03.012 15907473 PMC3351201

[B11] Hinton A. Bond S. Forgac M. (2009). V-ATPase functions in normal and disease processes. *Pflugers Arch.* 457 589–598. 10.1007/s00424-007-0382-4 18026982

[B12] Inoue T. Forgac M. (2005). Cysteine-mediated cross-linking indicates that subunit C of the V-ATPase is in close proximity to subunits E and G of the V1 domain and subunit a of the V0 domain. *J. Biol. Chem.* 280 27896–27903. 10.1074/jbc.m504890200 15951435

[B13] Inoue T. Wang Y. Jefferies K. Qi J. Hinton A. Forgac M. (2005). Structure and regulation of the V-ATPases. *J. Bioenerg. Biomembr.* 37 393–398. 16691471 10.1007/s10863-005-9478-8

[B14] Jianhua Z. Samir B. John L. R. (2015). Electron cryomicroscopy observation of rotational states in a eukaryotic V-ATPase. *Nature* 521 241–245. 10.1038/nature14365 25971514

[B15] Julian A. T. D. (1999). The multifunctional *Drosophila melanogaster* V-ATPase is encoded by a multigene family. *J. Bioenerg. Biomembr.* 31 75–83. 10340851 10.1023/a:1005400731289

[B16] Kobia F. Duchi S. Deflorian G. Vaccari T. (2013). Pharmacologic inhibition of vacuolar HC ATPase reduces physiologic and oncogenic Notch signaling. *Mol. Oncol.* 8 207–220. 10.1016/j.molonc.2013.11.002 24309677 PMC5528540

[B17] Krogh A. Larsson B. von Heijne G. Sonnhammer E. L. (2001). Predicting transmembrane protein topology with a hidden Markov model: application to complete genomes. *J. Mol. Biol.* 305 567–580. 10.1006/jmbi.2000.4315 11152613

[B18] Leng X. H. Manolson M. Forgac M. (1998). Function of the COOH-terminal domain of Vph1p in activity and assembly of the yeast V-ATPase. *J. Biol. Chem.* 273 6717–6723. 10.1074/jbc.273.12.6717 9506970

[B19] Leng X. H. Manolson M. Liu Q. Forgac M. (1996). Site-directed mutagenesis of the 100-kDa subunit (Vph1p) of the yeast vacuolar (H+□)-ATPase. *J. Biol. Chem.* 271 22487–22493. 10.1074/jbc.271.37.22487 8798414

[B20] Liégeois S. Benedetto A. Michaux G. Belliard G. Labouesse M. (2007). Genes required for osmoregulation and apical secretion in *Caenorhabditis elegans*. *Genetics* 175 709–724. 10.1534/genetics.106.066035 17179093 PMC1800596

[B21] Logan C. Y. Nusse R. (2004). The Wnt signaling pathway in development and disease. *Annu. Rev. Cell Dev. Biol.* 20 781–810. 15473860 10.1146/annurev.cellbio.20.010403.113126

[B22] Minh B. Q. Nguyen M. A. T. von Haeseler A. (2013). Ultrafast approximation for phylogenetic bootstrap. *Mol. Biol. Evol.* 30 1188–1195. 10.1093/molbev/mst024 23418397 PMC3670741

[B23] Mondragon A. A. Yalonetskaya A. Ortega A. J. Elguero J. Chung W. S. McCall K. (2019). Lysosomal machinery drives extracellular acidification to direct non- apoptotic cell death. *Cell Rep.* 27 11–19. 30943394 10.1016/j.celrep.2019.03.034 PMC6613820

[B24] Moussian B. (2010). Recent advances in understanding mechanisms of insect cuticle differentiation. *Insect. Biochem. Mol. Biol.* 40 363–375. 10.1016/j.ibmb.2010.03.003 20347980

[B25] Nagata R. Nakamura M. Sanaki Y. Igaki T. (2019). Cell competition is driven by autophagy. *Dev. Cell.* 51 99–112. 31543447 10.1016/j.devcel.2019.08.018

[B26] Nelson N. (2003). A journey from mammals to yeast with vacuolar H+-ATPase (V-ATPase). *J. Bioenerg. Biomembr.* 35 281–289. 14635774 10.1023/a:1025768529677

[B27] Nishi T. Forgac M. (2000). Molecular cloning and expression of three isoforms of the 100-kDa a subunit of the mouse vacuolar proton-translocating ATPase. *J. Biol. Chem.* 275 6824–6830. 10.1074/jbc.275.10.6824 10702241

[B28] Overend G. Luo Y. Henderson L. Douglas A. E. Davies S. A. Dow J. A. (2016). Molecular mechanism and functional significance of acid generation in the *Drosophila* midgut. *Sci. Rep.* 6:27242. 10.1038/srep27242 PMC4890030 27250760

[B29] Petzoldt A. G. Gleixner E. M. Fumagalli A. Vaccari T. Simons M. (2013). Elevated expression of the V-ATPase C subunit triggers JNK-dependent cell invasion and overgrowth in a *Drosophila* epithelium. *Dis. Model. Mech.* 6 689–700. 10.1242/dmm.010660 23335205 PMC3634652

[B30] Portela M. Yang L. Paul S. Li X. Veraksa A. Parsons L. M. (2018). Lgl reduces endosomal vesicle acidification and Notch signaling by promoting the interaction between Vap33 and the V-ATPase complex. *Sci. Signal.* 11:eaar1976. 10.1126/scisignal.aar1976 29871910 PMC6437781

[B31] Qi J. Wang Y. Forgac M. (2007). The vacuolar (H+)-ATPase: subunit arrangement and in vivo regulation. *J. Bioenerg. Biomembr.* 39 423–426. 10.1007/s10863-007-9116-8 18040762

[B32] Ren L. Mo D. Li Y. Liu T. Yin H. Jiang N. (2018). A genetic mosaic screen identifies genes modulating Notch signaling in *Drosophila*. *PLoS One* 13:e0203781. 10.1371/journal.pone.0203781 30235233 PMC6147428

[B33] Sarov M. Barz C. Jambor H. Hein M. Y. Schmied C. Suchold D. (2016). A genome-wide resource for the analysis of protein localisation in *Drosophila*. *eLife* 5:e12068. 10.7554/eLife.12068 PMC4805545 26896675

[B34] Sato A. Kojima T. Ui-Tei K. Miyata Y. Saigo K. (1999). Dfrizzled-3, a new *Drosophila* Wnt receptor, acting as an attenuator of Wingless signaling in wingless hypomorphic mutants. *Development* 126 4421–4430. 10498678 10.1242/dev.126.20.4421

[B35] Saw N. M. Kang S. Y. Parsaud L. Han G. A. Jiang T. Grzegorczyk K. (2011). Vacuolar H(+)-ATPase subunits Voa1 and Voa2 cooperatively regulate secretory vesicle acidification, transmitter uptake, and storage. *Mol. Biol. Cell.* 22 3394–3409. 10.1091/mbc.e11-02-0155 21795392 PMC3172264

[B36] Su Y. Ospina J. K. Zhang J. Michelson A. P. Schoen A. M. Zhu A. J. (2011). Sequential phosphorylation of smoothened transduces graded hedgehog signaling. *Sci. Signal.* 4:ra43. 10.1126/scisignal.2001747 21730325 PMC3526344

[B37] Swarup S. Verheyen E. M. (2012). Wnt/Wingless signaling in *Drosophila*. *Cold Spring Harb. Perspect. Biol.* 4:a007930. 10.1101/cshperspect.a007930 PMC3367557 22535229

[B38] Theodosiou N. A. Xu T. (1998). Use of FLP/FRT system to study *Drosophila* development. *Methods* 14 355–365. 10.1006/meth.1998.0591 9608507

[B39] Toei M. Saum R. Forgac M. (2010). Regulation and isoform function of the V-ATPases. *Biochemistry* 49 4715–4723. 10.1021/bi100397s 20450191 PMC2907102

[B40] Toyomura T. Murata Y. Yamamoto A. Oka T. Sun-Wada G. H. Wada Y. (2003). From lysosomes to the plasma membrane: localization of vacuolar-type H+ -ATPase with the a3 isoform during osteoclast differentiation. *J. Biol. Chem.* 278 22023–22030. 10.1074/jbc.m302436200 12672822

[B41] Toyomura T. Oka T. Yamaguchi C. Wada Y. Futai M. (2000). Three subunit a isoforms of mouse vacuolar H(+)-ATPase. *Preferential expression of the a*3 isoform during osteoclast differentiation. *J. Biol. Chem.* 275 8760–8765. 10.1074/jbc.275.12.8760 10722719

[B42] Trifinopoulos J. Nguyen L.-T. von Haeseler A. Minh B. Q. (2016). W-IQ-TREE: a fast online phylogenetic tool for maximum likelihood analysis. *Nucleic Acids Res.* 44 W232–W235. 27084950 10.1093/nar/gkw256 PMC4987875

[B43] Vaccari T. Duchi S. Cortese K. Tacchetti C. Bilder D. (2010). The vacuolar ATPase is required for physiological as well as pathological activation of the Notch receptor. *Development* 137 1825–1832. 10.1242/dev.045484 20460366 PMC2867318

[B44] Wang D. Epstein D. Khalaf O. Srinivasan S. Williamson W. R. Fayyazuddin A. (2014). Ca2+-Calmodulin regulates SNARE assembly and spontaneous neurotransmitter release via v-ATPase subunit V0 a1. *J. Cell Biol.* 205 21–31. 10.1083/jcb.201312109 24733584 PMC3987144

[B45] Wang J. Kean L. Yang J. Allan A. K. Davies S. A. Herzyk P. (2004). Function-informed transcriptome analysis of *Drosophila* renal tubule. *Genome Biol.* 5:R69. 10.1186/gb-2004-5-9-r69 PMC522876 15345053

[B46] Wang Y. Carballo R. G. Moussian B. (2017). Double cuticle barrier in two global pests, the whitefly *Trialeurodes vaporariorum* and the bedbug *Cimex lectularius*. *J. Exp. Biol.* 220 1396–1399. 10.1242/jeb.156679 28167802

[B47] Wang Y. Yu Z. Zhang J. Moussian B. (2016). Regionalization of surface lipids in insects. *Proc. Biol. Sci.* 283:20152994. 10.1098/rspb.2015.2994 27170708 PMC4874700

[B48] Wieczorek H. Beyenbach K. W. Huss M. Vitavska O. (2009). Vacuolar-type proton pumps in insect epithelia. *J. Exp. Biol.* 212 1611–1619. 10.1242/jeb.030007 19448071 PMC2683008

[B49] Wilkens S. Inoue T. Forgac M. (2004). Three-dimensional structure of the vacuolar ATPase: localization of subunit H by difference imaging and chemical cross-linking. *J. Biol. Chem.* 279 41942–41949. 10.1074/jbc.m407821200 15269204

[B50] Williamson W. R. Wang D. Haberman A. S. Hiesinger P. R. (2010a). A dual function of V0-ATPase a1 provides an endolysosomal degradation mechanism in *Drosophila melanogaster* photoreceptors. *J. Cell Biol.* 189 885–899. 10.1083/jcb.201003062 20513768 PMC2878941

[B51] Williamson W. R. Yang T. Terman J. R. Hiesinger P. R. (2010b). Guidance receptor degradation is required for neuronal connectivity in the *Drosophila* nervous system. *PLoS Biol.* 8:e1000553 10.1371/journal.pone.01000553 PMC2998435 21151882

[B52] Wissel S. Harzer H. Bonnay F. Burkard T. R. Neumüller R. A. Knoblich J. A. (2018). Time-resolved transcriptomics in neural stem cells identifies a v-ATPase/Notch regulatory loop. *J. Cell Biol.* 217 3285–3300. 10.1083/jcb.201711167 29959232 PMC6123005

[B53] Xu T. Rubin G. M. (1993). Analysis of genetic mosaics in developing and adult *Drosophila* tissues. *Development* 117 1223–1237. 8404527 10.1242/dev.117.4.1223

[B54] Yan Y. Denef N. Schucpbach T. (2009). The vacuolar proton pump, V- ATPase, is required for notch signaling and endosomal trafficking in *Drosophila*. *Dev. Cell.* 17 387–402. 10.1016/j.devcel.2009.07.001 19758563 PMC2758249

