# Supplementary figures and images for: Investigation of Isoform Specific Functions of the V-ATPase a Subunit During Drosophila Wing Development
Source: Front Genet. 2020 Jul 10;11:723. doi: 10.3389/fgene.2020.00723 (PMC7365883; doi:10.3389/fgene.2020.00723)

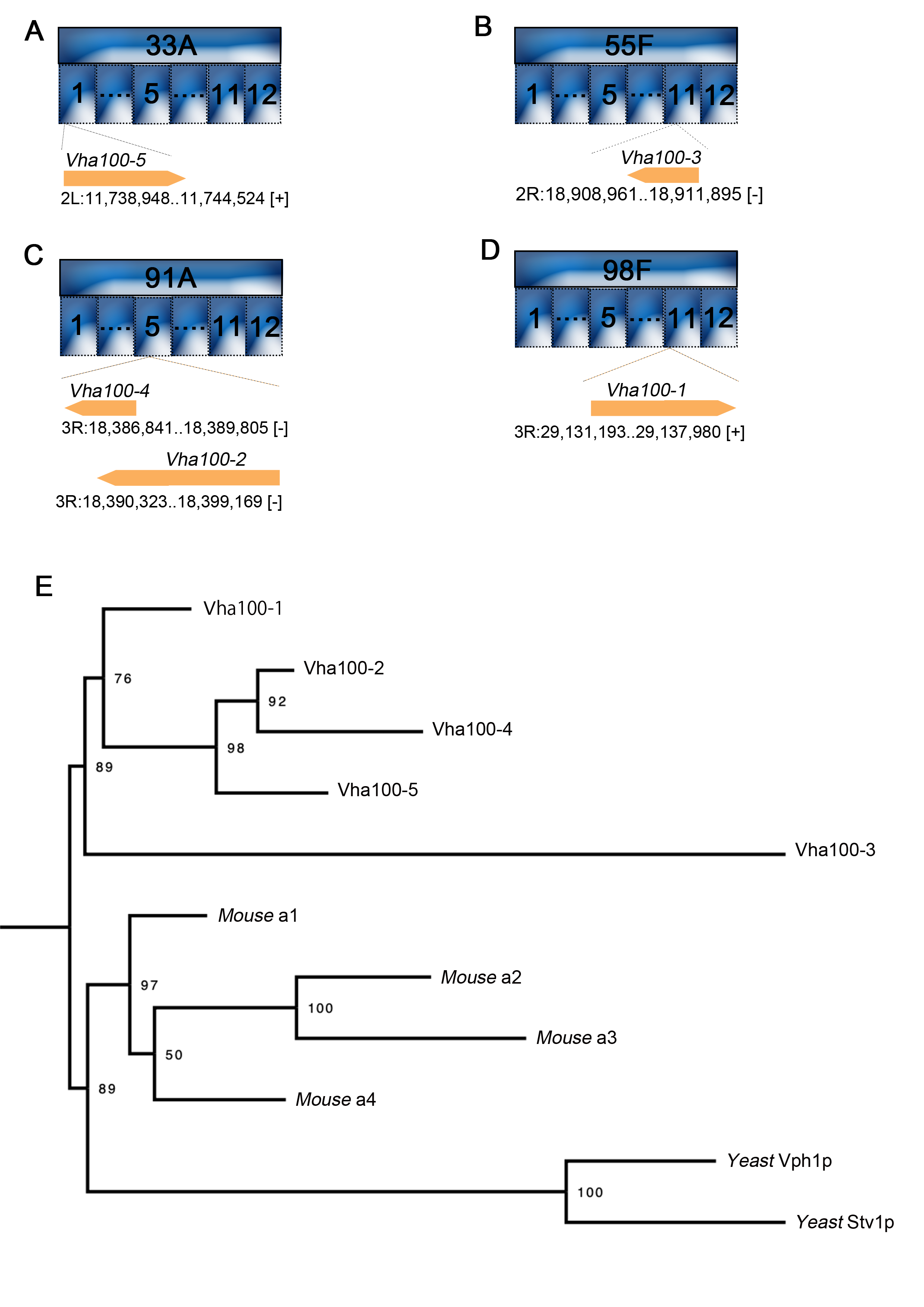

Supplement: Supplementary file 1 [file Image_1.TIF]

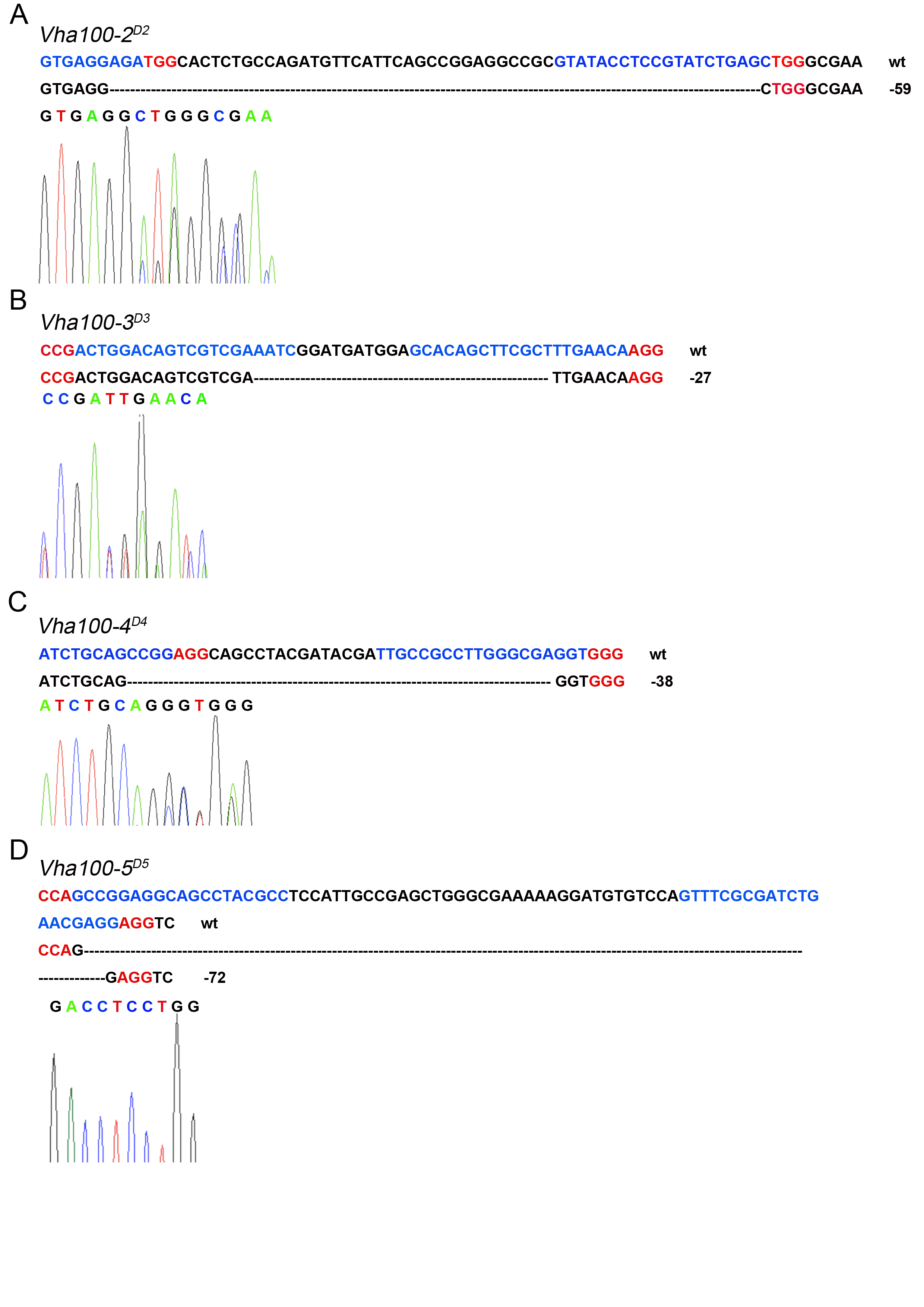

Supplement: Supplementary file 2 [file Image_2.TIF]

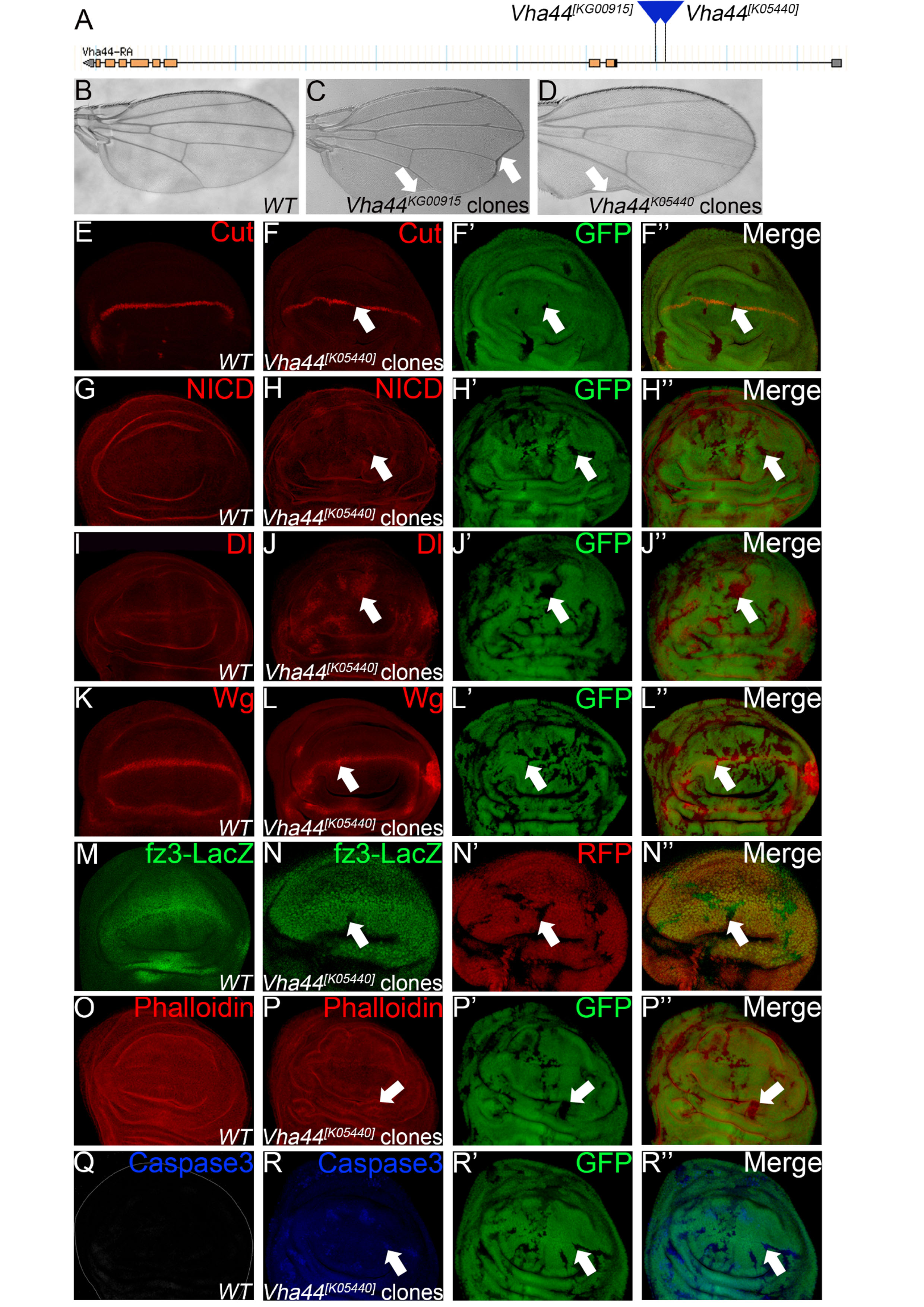

Supplement: Supplementary file 3 [file Image_3.JPEG]

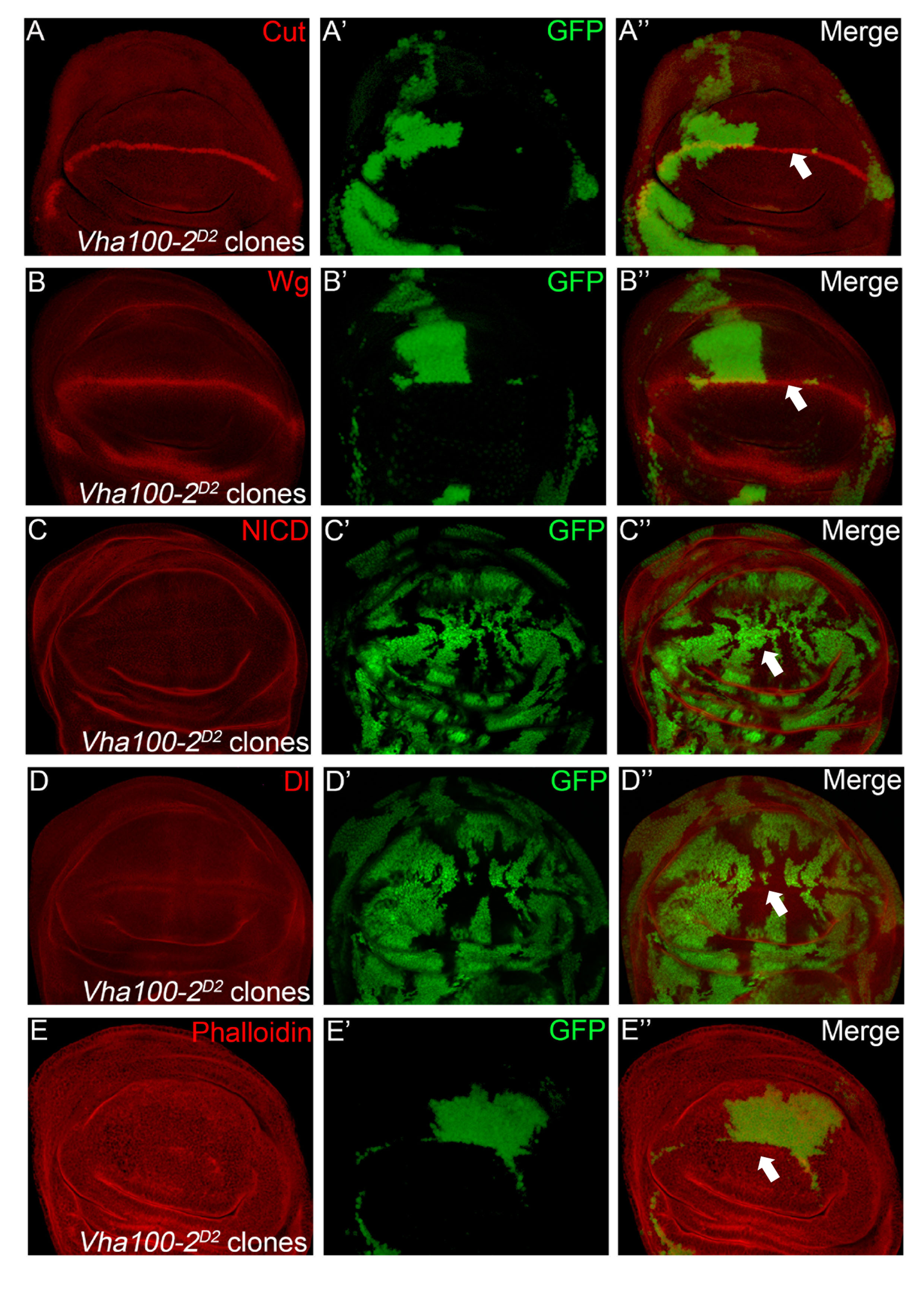

Supplement: Supplementary file 4 [file Image_4.JPEG]

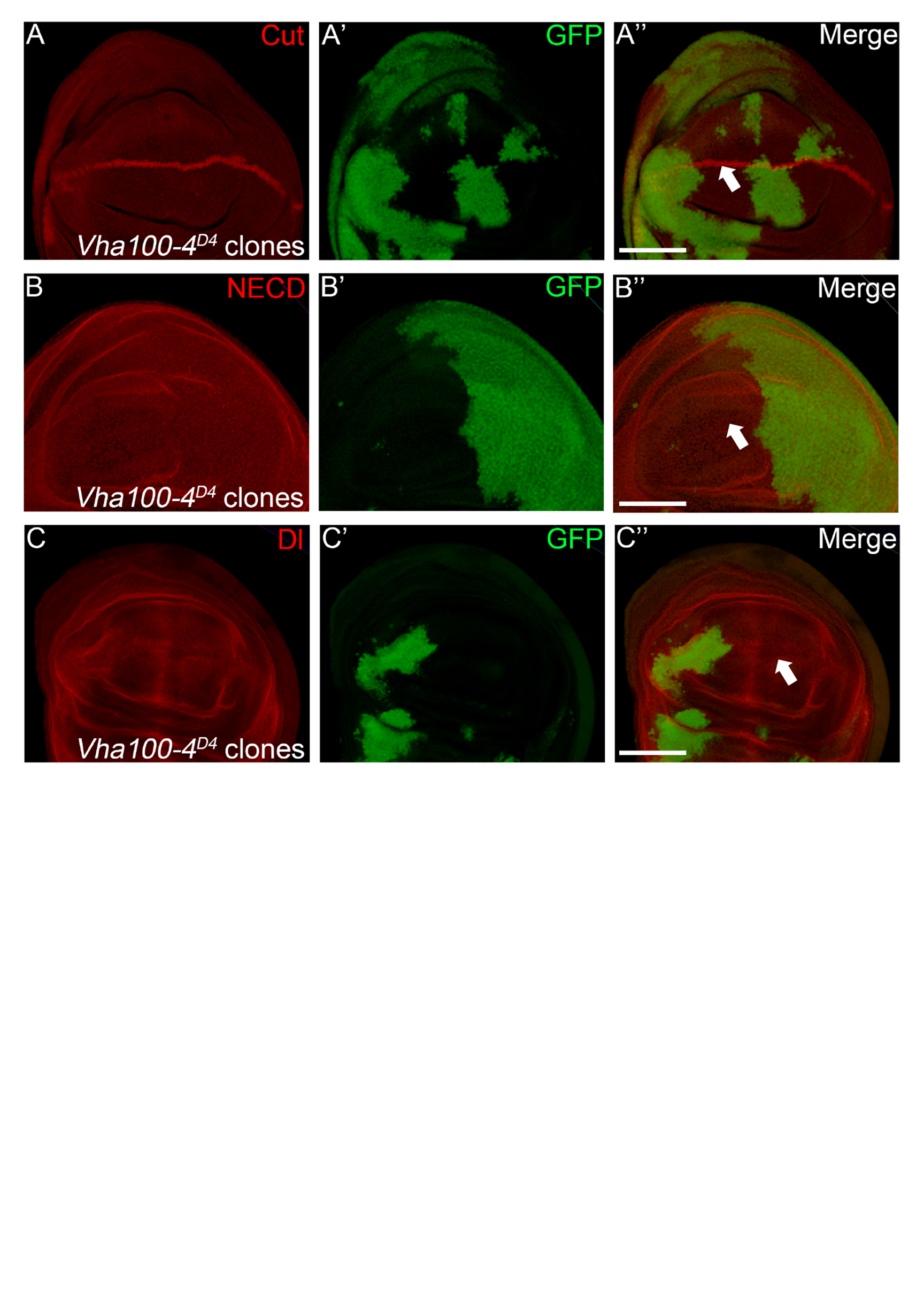

Supplement: Supplementary file 5 [file Image_5.TIF]

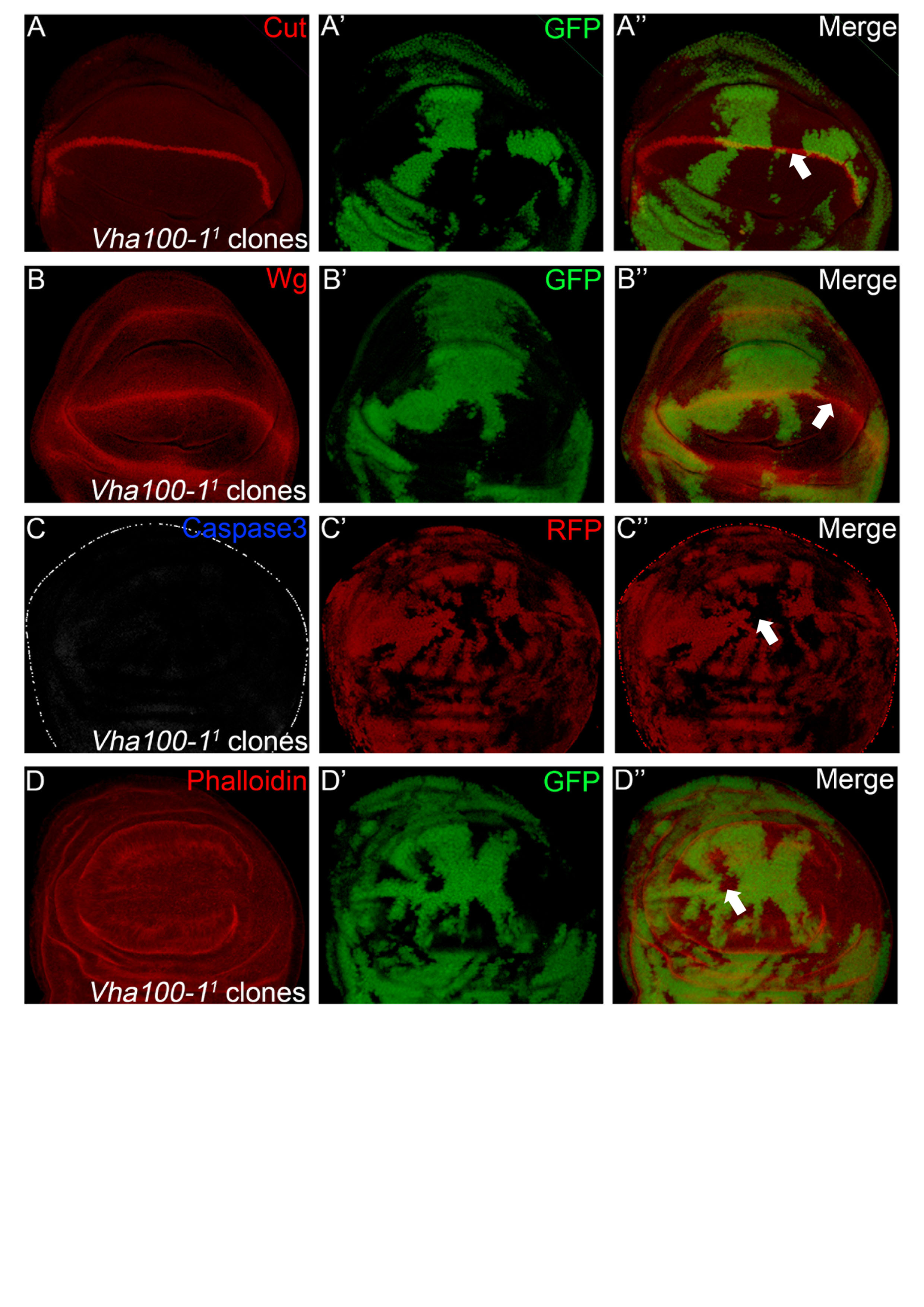

Supplement: Supplementary file 6 [file Image_6.JPEG]
